# A Delayed Seroma of a Facial Full-Thickness Skin Graft Donor Site: A Case Report

**DOI:** 10.7759/cureus.71147

**Published:** 2024-10-09

**Authors:** Renat Ahatov, Michael Adkison, Andrew Armenta, Sarah Rivera De Pena, Richard F Wagner

**Affiliations:** 1 Dermatology, University of Texas Medical Branch, Galveston, USA; 2 Medicine, University of Texas Medical Branch, Galveston, USA

**Keywords:** delayed seroma, full thickness skin graft, mohs surgery, seroma formation, skin graft donor site

## Abstract

Seromas are characterized as an accumulation of serous fluid beneath the skin, commonly occurring as a postoperative complication. Such formations can occur in dermatologic surgeries where the undermining and dissection of soft tissue create an empty cavity for fluid accumulation. When seromas develop, they usually do so at the wound closure site, within approximately a week after repair. In some cases, seromas have been shown to occur beyond the expected timeframe, coining the term “late seromas.” In this context, we present a case of a postoperative seroma noted 21 days after harvesting a full-thickness skin graft from the donor site in the preauricular region.

## Introduction

A seroma is an accumulation of serous fluid beneath the skin, often occurring as a postoperative complication [1]. The fluid typically collects in a surgical wound where an empty cavity has formed following extensive dissection of soft tissue. Seromas can present as soft, localized swellings that drain clear fluid and are either asymptomatic or accompanied by pain or functional deficits in the affected area. The etiology of seroma formation is believed to result from the disruption of blood and lymphatic vessels during soft tissue dissections, which leads to the leakage and accumulation of serous fluid into the dead space [2]. As such, seroma fluid typically consists of lymphatic fluid and plasma. Seromas can occur in dermatologic surgeries where undermining and dissection of soft tissue are necessary for wound closure [3]. In this context, we present a case of a postoperative seroma noted 21 days after the primary closure of a full-thickness skin graft donor site in the preauricular region.

## Case presentation

**Figure 1:**
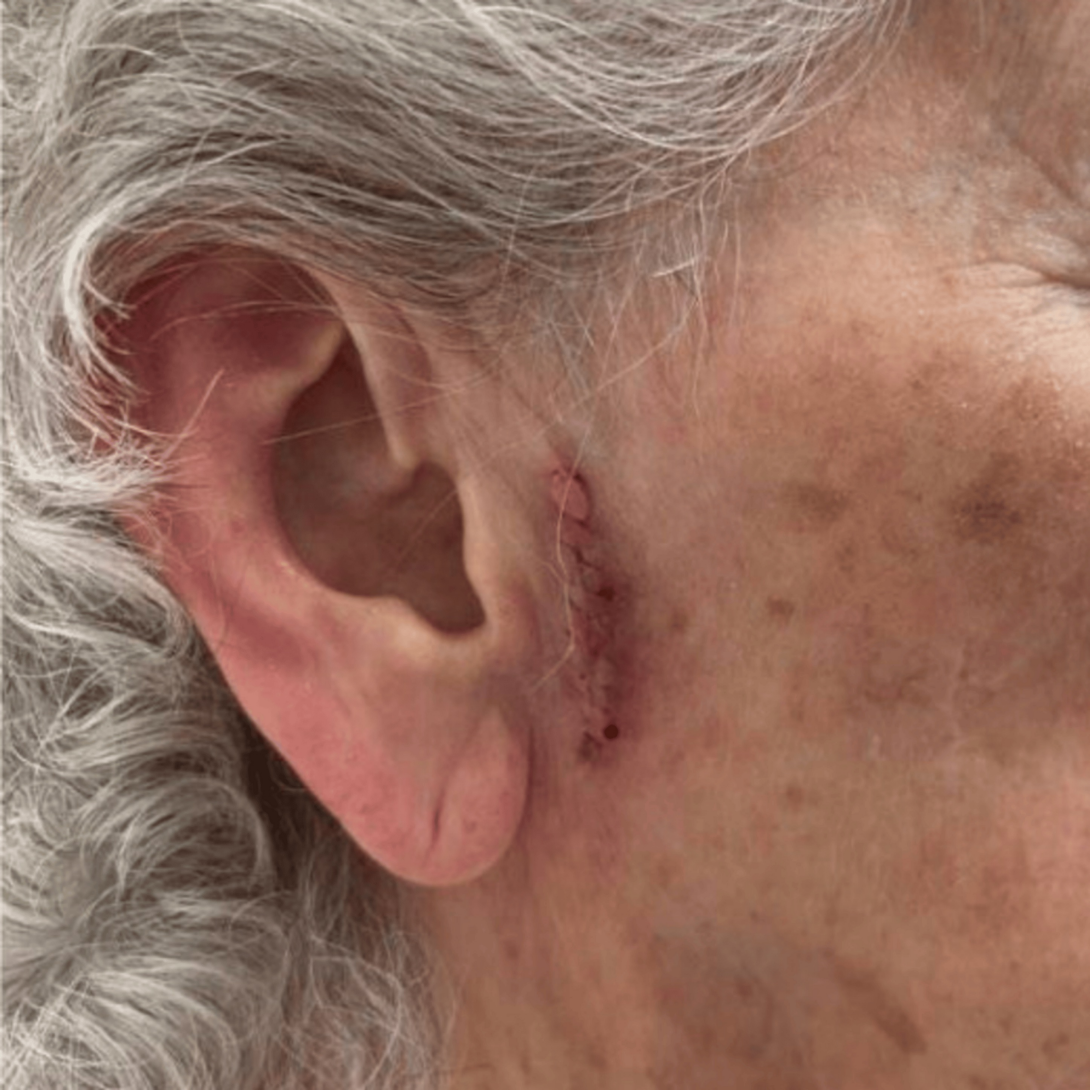
The graft donor site 14 days post-operation

**Figure 2:**
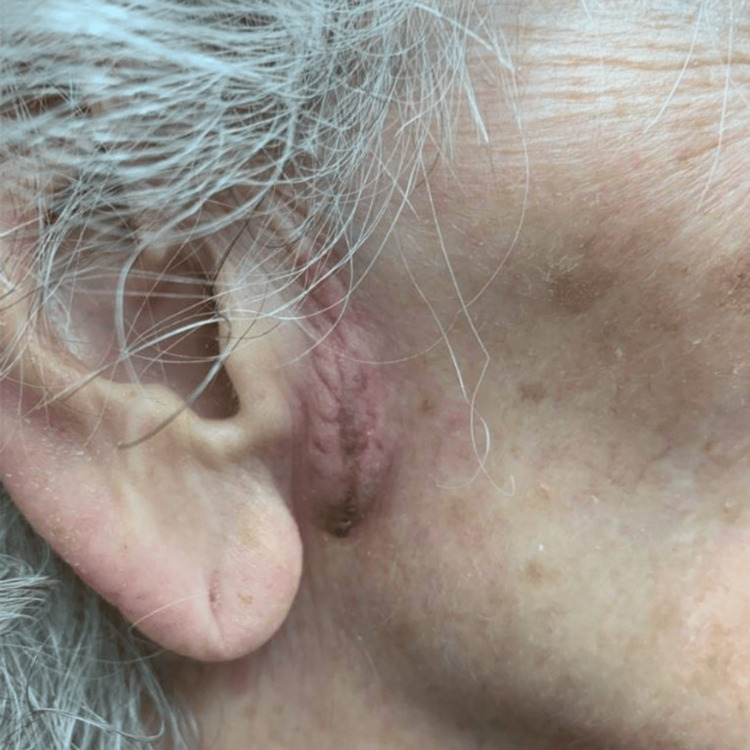
Seroma present 22 days post-operation

**Figure 3:**
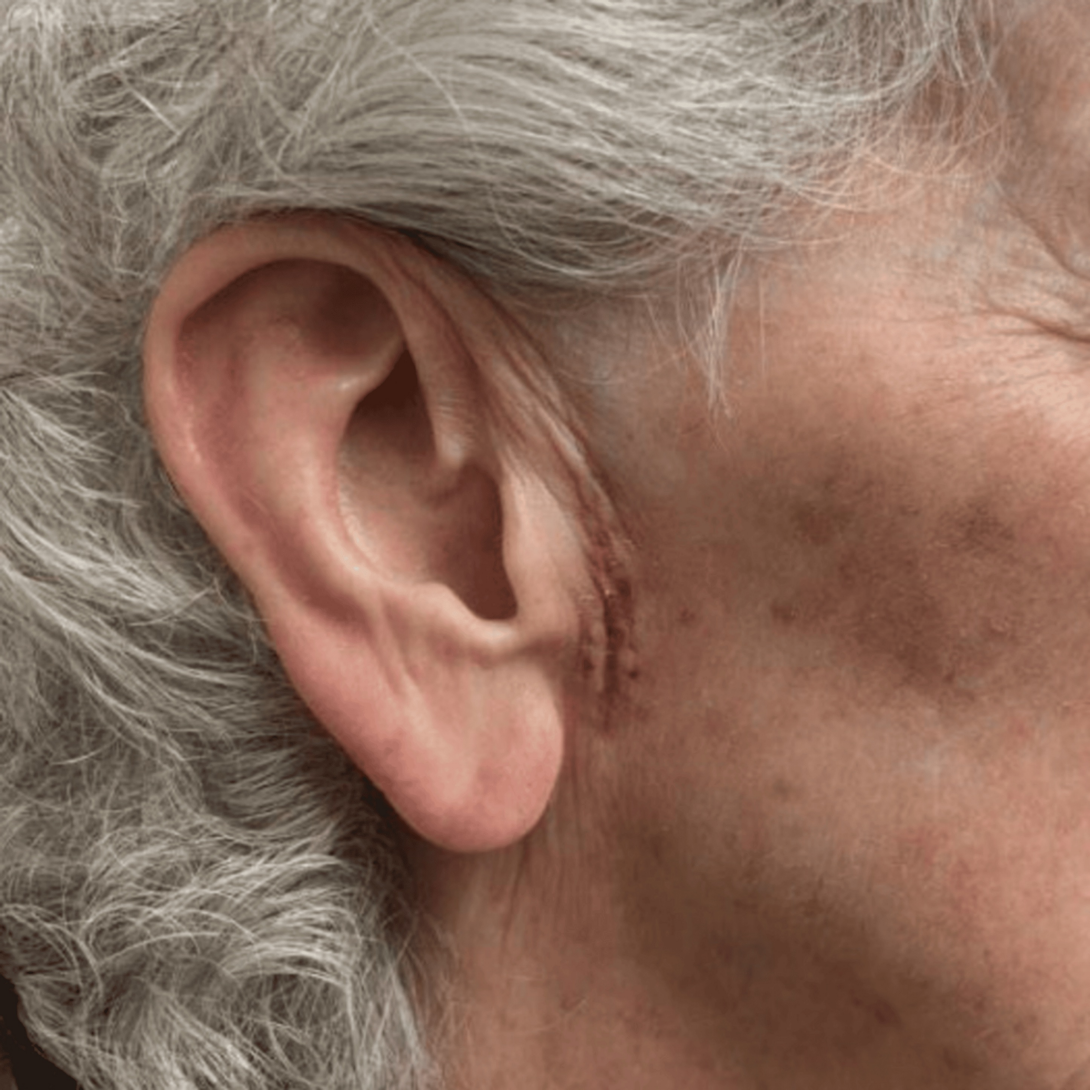
The graft donor site healing status-post seroma drainage 28 days post-operation

**Figure 4:**
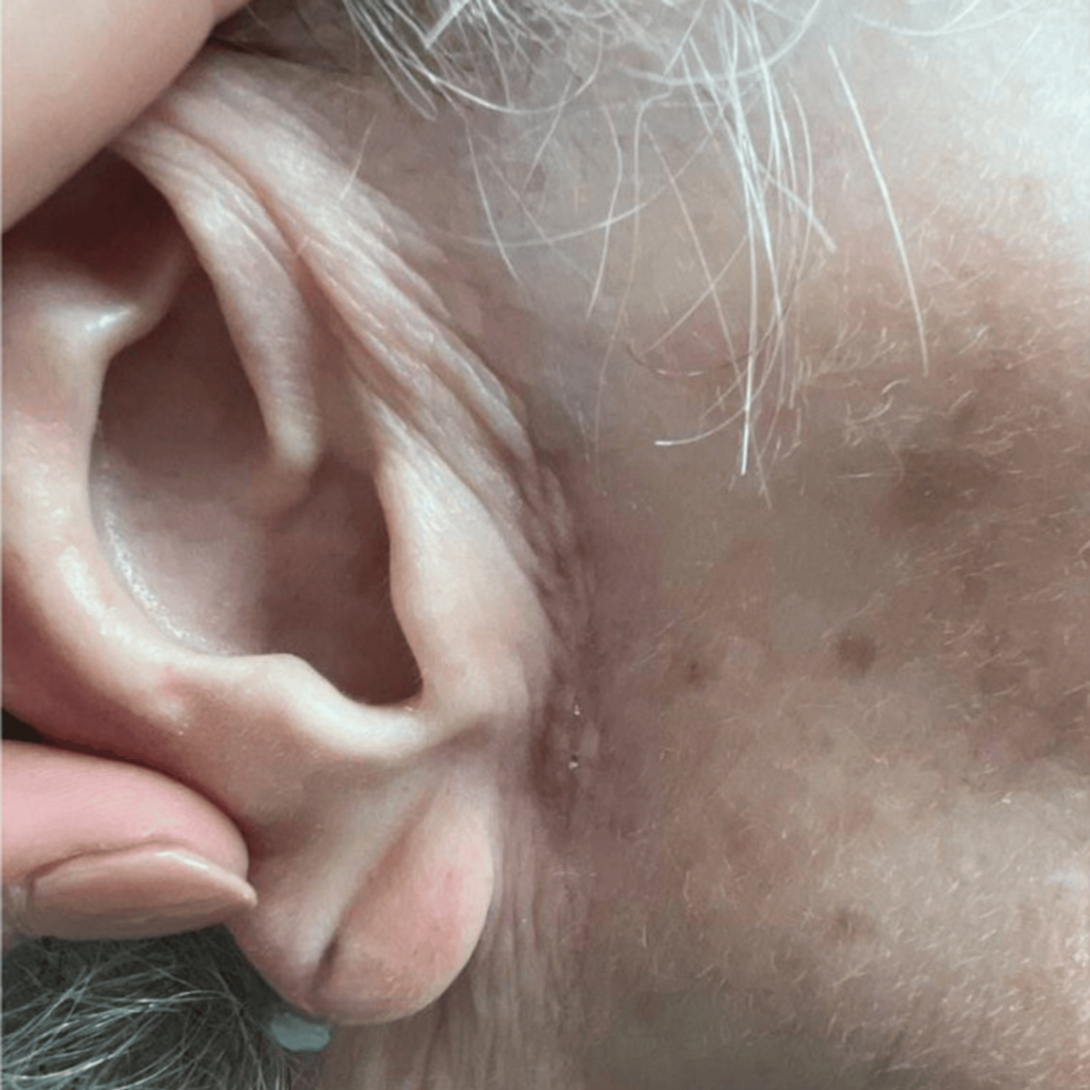
The graft donor site healing status-post seroma drainage 56 days post-operation

An 87-year-old woman with a history of multiple skin cancers presented to the clinic with an erythematous papule and telangiectasias on the left nasal tip. A diagnostic tangential skin biopsy was performed and showed a basal cell carcinoma. After discussion, the patient opted for treatment by Mohs micrographic surgery (MMS). Following tumor excision, a full-thickness skin graft was harvested from the preauricular region and sutured into the wound on the nasal tip. Following the graft placement, the recipient site was dressed with petrolatum ointment and secured with a bolster, while the donor site was closed by primary closure and covered with a pressure dressing. The patient was started on mupirocin ointment for local wound care. Post-operation, 14 days later, her sutures were removed, and a well-healing graft with adequate cosmesis and no seroma was noted at the skin graft donor site (Figure 1). Post-operation, 22 days later, the patient returned with concerns about a one-day history of tenderness and swelling at the inferior aspect of the graft donor site (onset at 21 days post-operation). Examination revealed an erythematous to purpuric, fluctuant swelling in the right preauricular region the day after onset, consistent with a seroma (Figure 2). The seroma was subsequently drained with an 18-gauge needle and bandaged, and a follow-up assessment (on days 28 and 56 post-operation) showed resolution with no fluid re-accumulation (Figures 3-4).

## Discussion

Seromas typically develop at the surgical incision site approximately 7 to 10 days following wound closure [1]. However, seromas have been shown to occur beyond this timeframe in breast implant and reconstruction surgeries, termed late seromas. These seromas were found to appear months to years following the surgeries [4]. To our knowledge, no cases in dermatologic surgery have described a late-onset seroma. In our case, seroma formation was noted 21 days following wound closure. The exact etiology of delayed seromas is not well understood, although one report suspects a multifactorial origin, possibly involving trauma or subclinical infection [4]. In dermatologic surgery, seromas have been shown to develop following skin grafting due to dead space created from tissue removal, poor wound healing, or lymphatic vessel damage. Notably, in one case series involving six cases requiring skin grafting, seroma formation was observed beneath the graft at the recipient site [5]. Conversely, in the case we are presenting, seroma formation was later than commonly seen and was observed at the skin graft donor site. Diagnosis of seromas is typically made clinically, with characteristic features including localized swelling and clear fluid drainage. Complications include wound dehiscence, abscess, and pseudocyst formation, as well as flap necrosis or skin graft failure [1,2,5]. Typically, the body will reabsorb the seroma fluid without requiring further management; however, needle aspiration and antibiotic therapy may be warranted if the seroma persists or shows signs of infection.

## Conclusions

To our knowledge, this is the first report of its kind to describe a late-onset seroma at the graft donor site following MMS. This case highlights that seromas may be considered the underlying cause of localized postoperative swelling at the surgical or donor graft site, even when occurring beyond the typical 7-to-10-day timeframe. In conclusion, we present a case that demonstrates an atypical presentation of a delayed-onset seroma at the donor site of a full-thickness skin graft. Future studies may aim to further investigate the underlying etiology of late-onset seromas.
